# T Cell Intrinsic NOD2 Is Dispensable for CD8 T Cell Immunity

**DOI:** 10.1371/journal.pone.0056014

**Published:** 2013-02-06

**Authors:** Gloria H. Y. Lin, Michael E. Wortzman, Stephen E. Girardin, Dana J. Philpott, Tania H. Watts

**Affiliations:** 1 Department of Immunology, University of Toronto, Toronto, Ontario, Canada; 2 Department of Laboratory Medicine and Pathobiology, University of Toronto, Toronto, Ontario, Canada; Saint Louis University School of Medicine, United States of America

## Abstract

NOD2 is an intracellular pattern recognition receptor that provides innate sensing of bacterial muramyl dipeptide by host cells, such as dendritic cells, macrophages and epithelial cells. While NOD2's role as an innate pathogen sensor is well established, NOD2 is also expressed at low levels in T cells and there are conflicting data as to whether NOD2 plays an intrinsic role in T cell function. Here we show that following adoptive transfer into WT hosts, NOD2^−/−^ OT-I T cells show a small decrease in the number of OVA-specific CD8 T cells recovered at the peak of the response to respiratory influenza virus infection. On the other hand, no such defect was observed upon intranasal immunization with a replication defective adenovirus carrying the OVA epitope recognized by OT-I, or when OVA was delivered with LPS subcutaneously, or when influenza-OVA was delivered intraperitoneally. Thus we observed a selective defect in NOD2-deficient T cell responses only during a live viral infection. Moreover, there was no apparent defect when NOD2^−/−^ OT-I T cells were stimulated *in vitro*. Finally, this selective defect in recovery of NOD2-deficient CD8 T cells was not observed in a non-transgenic respiratory infection model in which mixed bone marrow chimeras were used such that the NOD2^−/−^ T cells were allowed to develop and respond in a NOD2-sufficient host. Taken together our data indicate that T cell intrinsic NOD2 is not required for CD8 T cell responses to antigen delivered under a variety of conditions *in vitro* and *in vivo*. However, CD8 T cells that have developed in the absence of NOD2 show a selective and modest impairment in their response to live respiratory influenza infection.

## Introduction

NOD2, a member of the NLR family of leucine rich repeat proteins, is expressed in epithelial cells, macrophages and dendritic cells, where it functions as an innate pathogen sensor with specificity for bacterial muramyl dipeptide (MDP) [1]–[3]. NOD2 is of interest in human disease, as mutations in *NOD2* are associated with Crohn's disease, Blau syndrome and early onset sarcoidosis [4]. The effect of *NOD2* mutations is thought to relate to its role in the regulation of innate immune responses to pathogens as well as to constituents of the normal microbiota [5]. However, NOD2 is also expressed at low levels in T cells [6], raising the possibility that *NOD2* mutations could also have intrinsic effects on T cell responses. Indeed, an initial report suggested that T cell intrinsic NOD2 was important for CD4 T cell-dependent IFNγ responses to control *Toxiplasma gondii* in mice as well as for induction of colitis in an adoptive transfer model in which WT or NOD2^−/−^ CD4 T cells were transferred into RAG^−/−^ mice [7]. However, a follow-up study failed to confirm the requirement for NOD2 in CD4 T cell activation or for control of *T. gondii* [6]. Here we further address the role of NOD2 in T cells, focusing on the CD8 T cell response and show that CD8 intrinsic NOD2 is dispensable for their response in vitro and in vivo. However, T cells that have developed in the absence of NOD2 have a small and selective defect in their accumulation in vivo during respiratory influenza virus infection.

## Materials and Methods

### Ethics statement

All mouse experiments were conducted as approved by the University of Toronto animal care committee in accordance with the regulations of the Canadian Council on animal care (University of Toronto approved protocol #20009458).

### Mice

C57BL/6 wild type mice were obtained from Charles River Laboratories (St. Constant, QC, Canada). NOD2^−/−^ mice expressing EGFP under the control of the NOD2 promoter on the C57BL/6 were previously described [8] and were further crossed to generate NOD2^−/−^ OT-I mice. OT-I and CD45.1 congenic mice were obtained from Jackson Laboratories (Bar Harbor, Maine, USA) and crossed to generate CD45.1^+/+^ or OT-I mice. TCRα^−/−^ mice were kindly provided by Dr. Cynthia Guidos (Hospital for Sick Children, Toronto). Mice were maintained under specific pathogen free conditions in sterile microisolators at the University of Toronto. All mouse experiments were approved by the University of Toronto animal care committee in accordance with the regulations of the Canadian Council on animal care (University of Toronto approved protocol #20009458).

### Influenza virus infection

Influenza A/HKx31 and A/X31-OVA viruses were grown in eggs and their tissue culture infectious dose determined by infection of MDCK cells [9]. Age and sex-matched mice were used in all experiments. For intranasal infection, age- and sex- matched mice between 6–10 weeks of age were anaesthetized with isoflurane. 30 µL of diluted virus was given via nares. A dose of 5 HAU influenza A/X31 or 2.5 HAU of influenza A/X31-OVA was used for primary intranasal infection. For intraperitoneal influenza A/X31-OVA infection, virus was diluted in PBS and was injected in a 200 µL volume at a dose of 100 HAU.

### Adenoviral vectors and infection

Replication defective adenovirus 5 expressing the OT-I epitope, OVA257-264 in the E1 region (AdV-OVA) [10] was kindly provided by J. Bramson (McMaster University, Hamilton, ON, Canada). A dose of 10^9^ PFU of AdV-OVA was used for intranasal immunization.

### In vitro stimulation

Purified CD8 T cells from WT OT-I and NOD2^−/−^ OT-I mice were labeled with CFSE followed by stimulation irradiated WT C57BL/6 splenocytes that were pulsed with 10^−12^–10^−9^ M of OVA peptide at 1∶1 ratio. The CFSE signals were analyzed 2 days later. Purified WT and NOD2^−/−^ CD8 T cells were stimulated with plate bound anti-CD3 (1 µg/mL) with or without anti-CD28 (10 µg/mL) for two days, followed by analysis of cell surface markers.

### Immunization s.c. with OVA/LPS

One day following adoptive transfer of 10^4^ OT-I T cells, WT mice were injected subcutaneously with 1.5 mg of ovalbumin protein mixed with 37.5 µg of LPS in a volume of 200 µL.

### Bone marrow chimeras

Mixed bone marrow chimeras in which only the αβ T cells lack NOD2 were generated by reconstituting lethally irradiated congenically marked CD45.1^+^ mice with a 4∶1 mixture of TCRα^−/−^ bone marrow cells and NOD2^−/−^ bone marrow cells in a total of 5×10^6^ cells. This ratio was chosen to avoid a large decrease in the number of non-T cells lacking NOD, as the T cells expand homeostatically to compensate for the lower numbers of precursors. All irradiated, bone marrow reconstituted mice were given water supplemented with 2 mg/mL of neomycin sulfate (Bio-Shop, Burlington, Ontario, Canada) during the first four weeks, and they were further rested for an additional 2 months before analysis.

### CD8 T cell purification

OT-I T cells were purified from spleens of unimmunized naïve mice using a negative selection CD8 T cell enrichment kit (StemCell Technologies), and 10^4^ OT-I T cells were adoptively transferred to naïve WT host a day before infection or immunization.

### Flow cytometry and antibodies

Congenically marked OT-I TCR transgenic cells were tracked by using PE- or APC-anti-mouse CD45.1 (eBioscience), Pacific Blue-anti-CD45.1, Pacific Blue-anti-CD45.2 (Biolegend), PerCP-anti-mouse CD8 (BD Biosciences). Additional antibodies used in this study included PE-anti-CD69, APC-anti-CD62L, PE-anti-CD27, PE-anti-GITR, PE-anti-CD127, PE-anti-CD122, APC-anti-CCR7, APC-anti-IFNAR, APC-anti-KLRG-1, biotinylated anti-4-1BB, APC-anti-IFNγ, FITC-anti-CD107a, PE- or PE-Cy5- or APC-anti-CD44, Pe-Cy7- or PE-anti-mouse CD3. Samples were analyzed using FACScalibur, FACSCanto, or LSR II (BD Biosciences) with CellQuest or FACSDiva acquisition software. Data analysis was done using FlowJo software (TreeStar Inc., Ashland, OR, USA).

### Statistical analysis

Student's t-test (unpaired, two tailed, 95% confidence interval) was used for comparison of two groups. *, p<0.05; **, p<0.0; ***, p<0.001.

## Results

### Decreased recovery of NOD2^−/−^ TCR transgenic CD8 T cells following respiratory influenza infection in WT hosts

**Figure 1:**
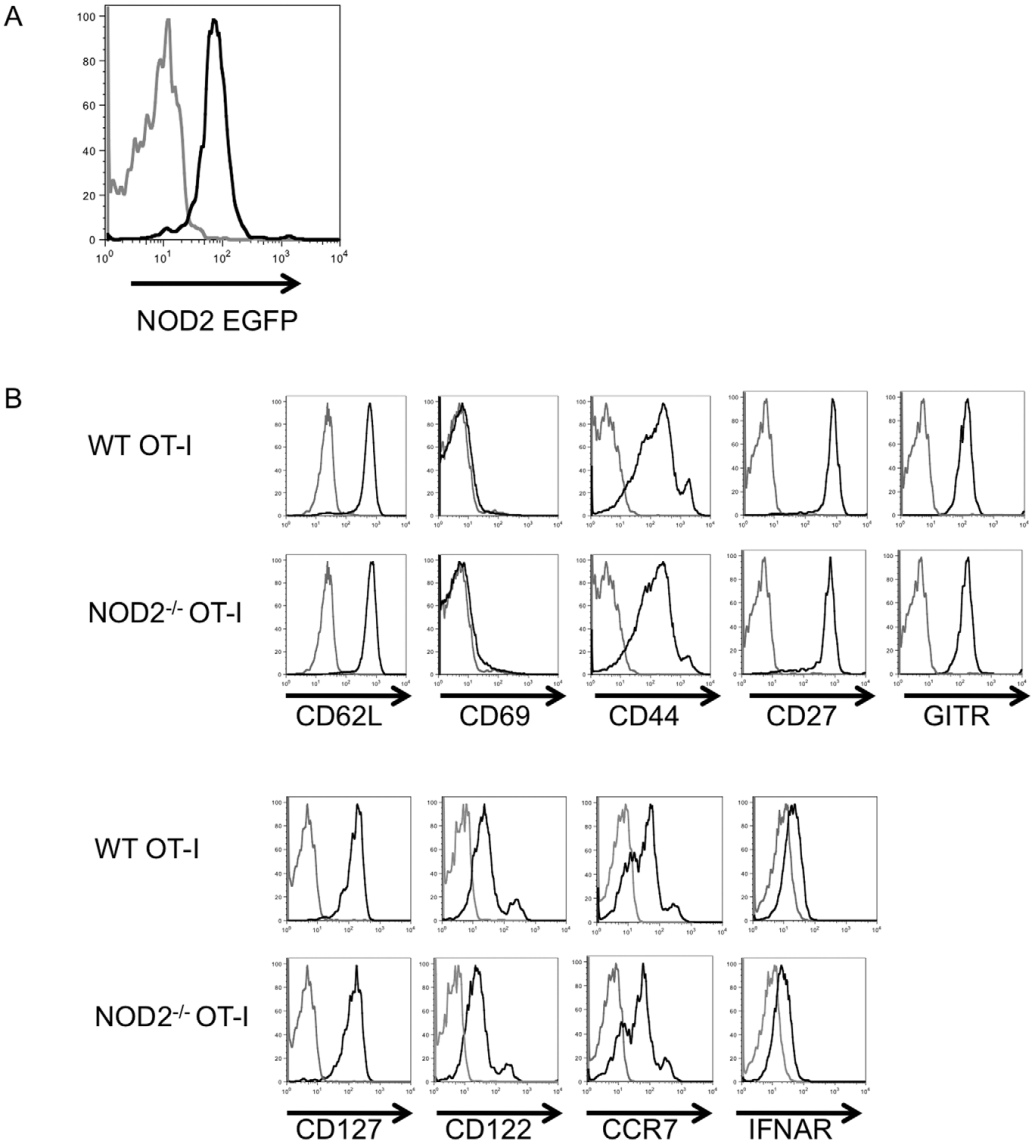
NOD2 deficient TCR Transgenic CD8 T cells exhibit a normal surface phenotype. (A) Expression of NOD2 on naïve OT-I CD8 T cells was analyzed using the EGFP reporter signal. The black line on the histogram represents the EGFP signal, and the gray line represent the signal obtained from mice without the EGFP reporter. (B) Surface Expression of CD62L, CD69, CD44, CD27, GITR, CD127, CD122, and CCR7 were analyzed on splenic CD8 T cells obtained from unimmunized 6–8 weeks old WT or NOD2^−/−^ OT-I mice. Results shown are gated on the CD8^+^ T cells. The black line on the histogram represents the stain for the respective molecule, and the grey line represents fluorescence minus one control. Results in A and B are representative of two experiments, each with 2 mice per group.

To analyze the role of T cell intrinsic NOD2 protein in the immune response to influenza virus, we crossed CD45.1 OT-I TCR transgenic mice with NOD2-deficient mice that express an EGFP reporter under control of the NOD2 promoter [8]. We confirmed that NOD2 is expressed in the purified. OT-I T cells, based on EGFP reporter expression (Fig. 1A). The absence of NOD2 did not affect the recovery of naïve OT-I T cells from the mice (data not shown). Moreover, the naïve CD8 T cells isolated from WT or NOD2^−/−^ OT-I mice had an indistinguishable phenotype with respect to the cell surface expression of CD62L, CD69, CD44, CD27, GITR, CD127, CD122, CCR7 and the type I IFN receptor IFNAR (Fig. 1B).

**Figure 2:**
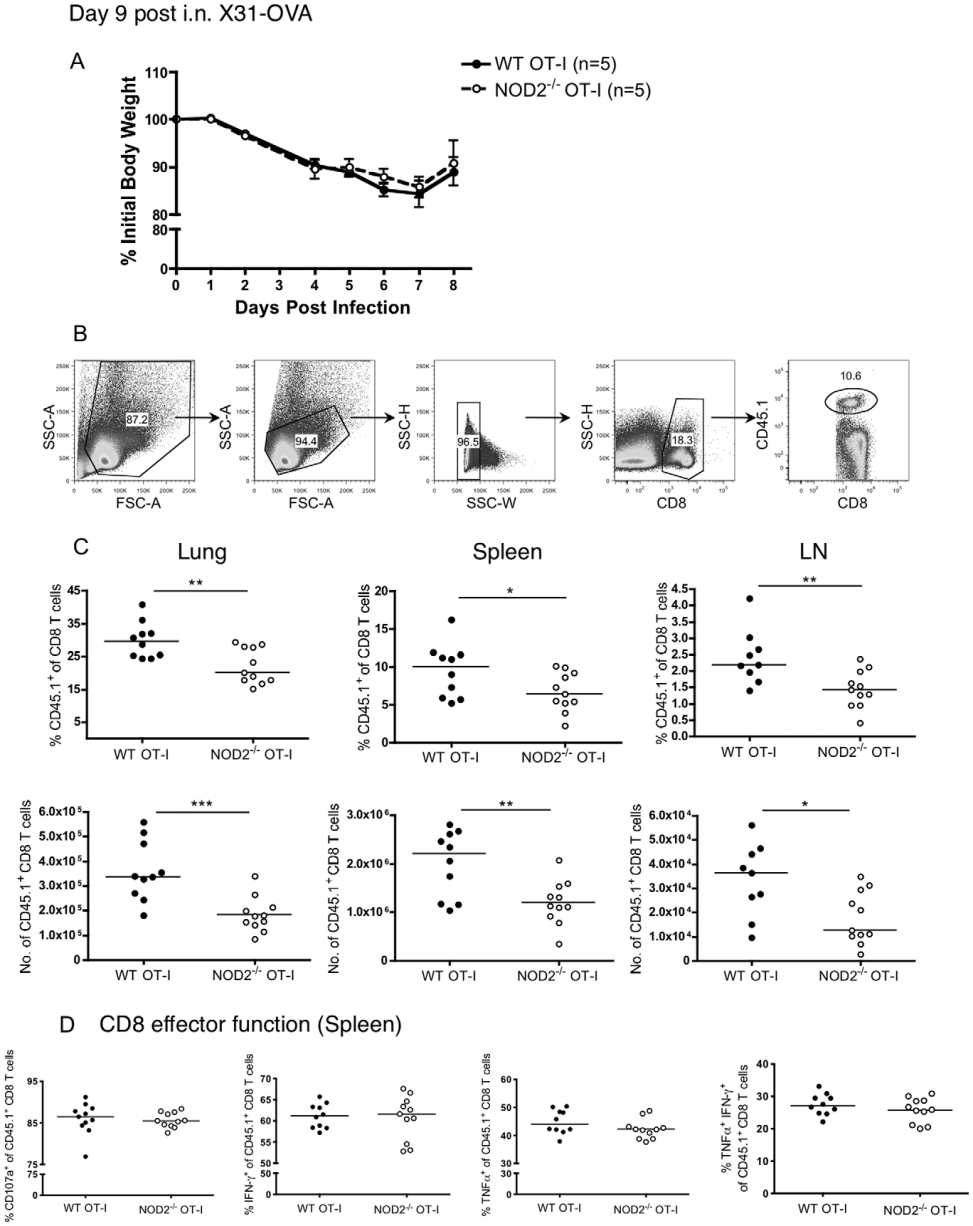
Decreased recovery of NOD2^−/−^ compared to WT TCR transgenic CD8 T cells following respiratory influenza infection of mice. 10^4^ purified CD8 T cells from CD45.1^+^ WT or CD45.1^+^ NOD2^−/−^ OT-I mice were injected i.v. into naïve WT CD45.2 mice, and one day later mice were intranasally infected with Influenza A/X31-OVA. (A) Body weights of the mice following influenza infections are shown as mean ± SEM for 5 mice per group. On day 9, mice were sacrificed and the percent (B, C) of OT-I out of the CD8 T cell population as well as the total number of OT-I T cells recovered from the lungs, spleens and mediastinal lymph nodes were analyzed (C). (D) The effector function of the transferred OT-I T cells were analyzed by CD107a, TNFα, and IFNγ staining following a 5-hour restimulation with 1 µM SIINFEKL peptide. The data in A are representative of two independent experiments and the results in C and D show the combined results of two independent experiments, each with 5 mice per group.

**Figure 3:**
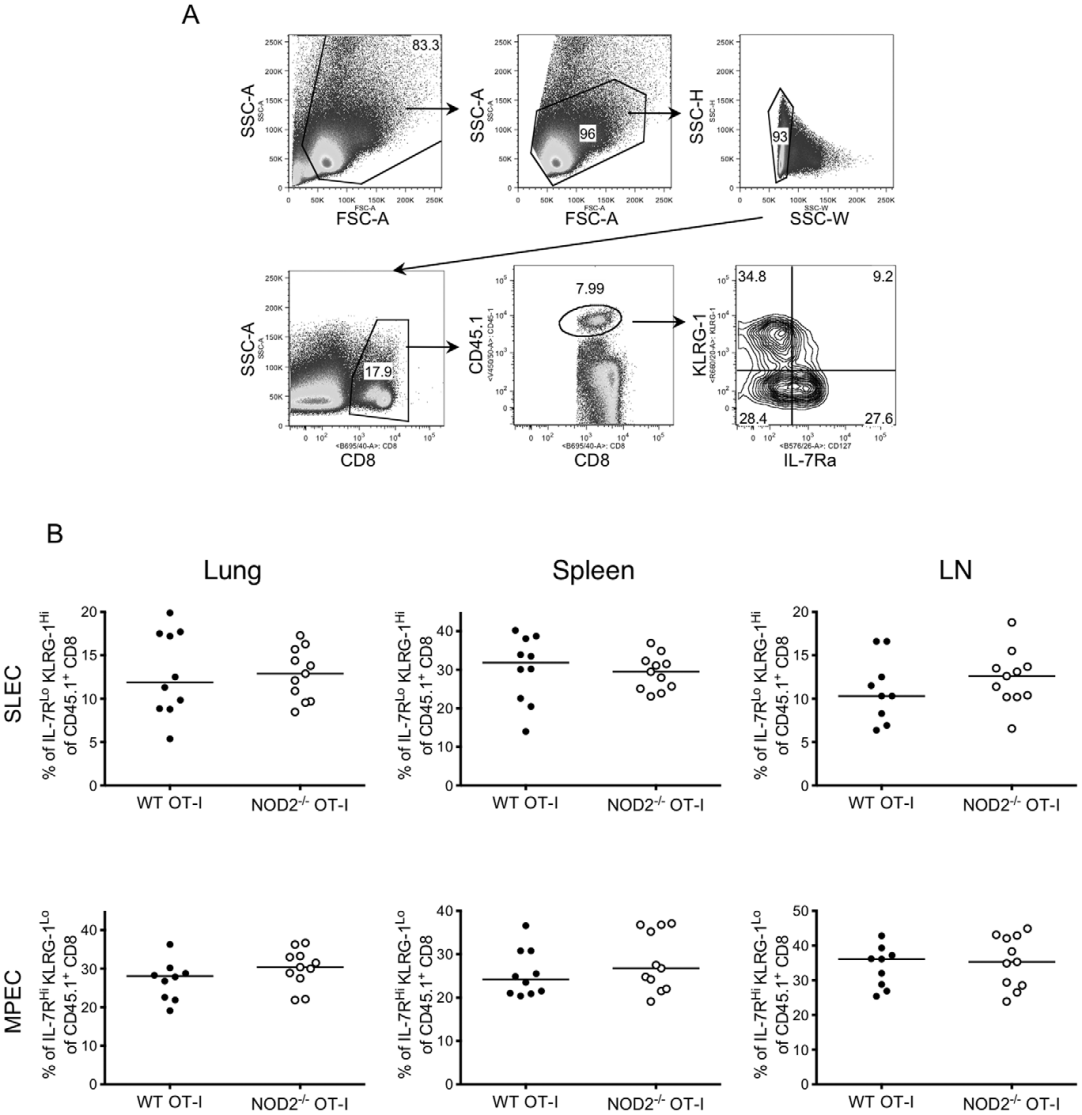
NOD2 deficient TCR Transgenic CD8 T cells have a normal percentage of IL-7α^Lo^ KLRG-1^Hi^ and IL-7α^Hi^ KLRG-1^Lo^ cells at the peak of respiratory influenza infection. 10^4^ purified CD8 T cells from CD45.1^+^ WT or CD45.1^+^ NOD2^−/−^ OT-I mice were injected i.v. into naïve WT mice, and one day later mice were intranasally infected with Influenza A/X31-OVA as in Figure 1. On day 9, mice were sacrificed and the percent of SLEC (IL-7Rα^Lo^ KLRG-1^Hi^) and MPEC (IL-7Rα^Hi^ KLRG-1^Lo^) were analyzed on CD45.1^+^ OT-I recovered from the lungs, spleens and mediastinal lymph nodes (A, B). The data in A and B show the combined results of two independent experiments, each with 5 mice per group.

To analyze the intrinsic role of NOD2 in CD8 T cells in vivo, we transferred 10^4^ purified congenically marked WT or NOD2^−/−^ OT-I CD8 T cells into naïve WT recipients. One day later, mice were infected intranasally with a recombinant influenza virus X31-OVA, carrying the SIINFEKL epitope recognized by the OT-I TCR in their neuraminidase stalk [11]. Mice that received WT or NOD2^−/−^ OT-I CD8 T cells showed similar weight loss in response to infection (Fig. 2A). However, at the peak of the T cell response (day 9 lung), there was a lower frequency and a 2-fold lower number of NOD2-deficient OT-I T cells recovered compared to WT OT-I T cells in lung, spleen and LN (Fig. 2C). Nevertheless, after peptide stimulation, the OT-I T cells recovered from the mice had a similar level of effector function per cell, regardless of their expression of NOD2 (Fig. 2D). Moreover, the WT and NOD2^−/−^ OT-I T cells at day 9 post-infection had indistinguishable levels of CD127 expression (data not shown). We also examined the percentages of short lived effector cells (SLECs, IL-7α^Lo^ KLRG-1^Hi^) and memory precursor effector cells (MPECs. IL-7Rα^Hi^ KLRG-1^Lo^) within the OT-I T cell pool at the peak of the response and found no difference in the differentiation into these two populations when the cells lack NOD2 (Fig. 3). This result shows that the absence of NOD2 affects the number of OT-I T cells that accumulate over the first 9 days of respiratory influenza infection, but does not affect the effector function or surface phenotype of the cells.

### T cells with or without NOD2 show similar responses in vitro as well as to s.c. immunization with OVA/LPS, i.p. immunization with influenza-X31-OVA or to replication defective adenovirus-OVA delivered i.n

**Figure 4:**
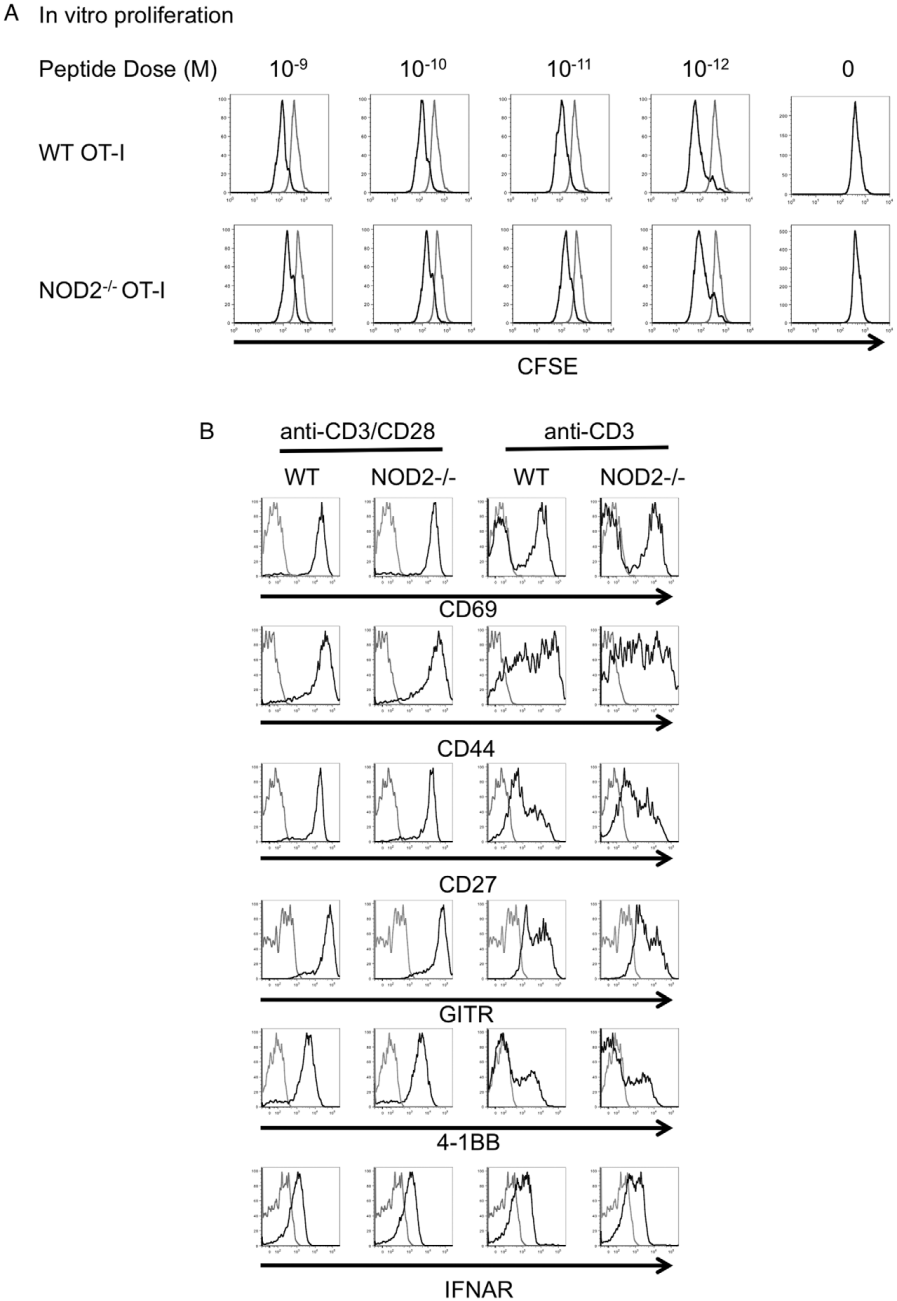
NOD2-deficient T cells divide normally in response to peptide antigen in vitro and have normal expression of surface marker following activation in vitro. (A) Purified CD8 T cells from WT OT-I and NOD2^−/−^ OT-I mice were CFSE labeled and then cultured with SIINFEKL-pulsed irradiated WT splenocytes. Two days later, the CFSE signals were analyzed on the OT-I T cells by gating on the live CD45.1^+^ OT-I T cells. Data are representative of two independent experiments. (B) Purified CD8 T cells from WT and NOD2^−/−^ spleens were stimulated with anti-CD3 with or without anti-CD28 for two days, followed by analysis of expression of activation markers, TNF receptor family members and IFNAR. The results are representative of three individual mice analyzed separately in a single experiment.

**Figure 5:**
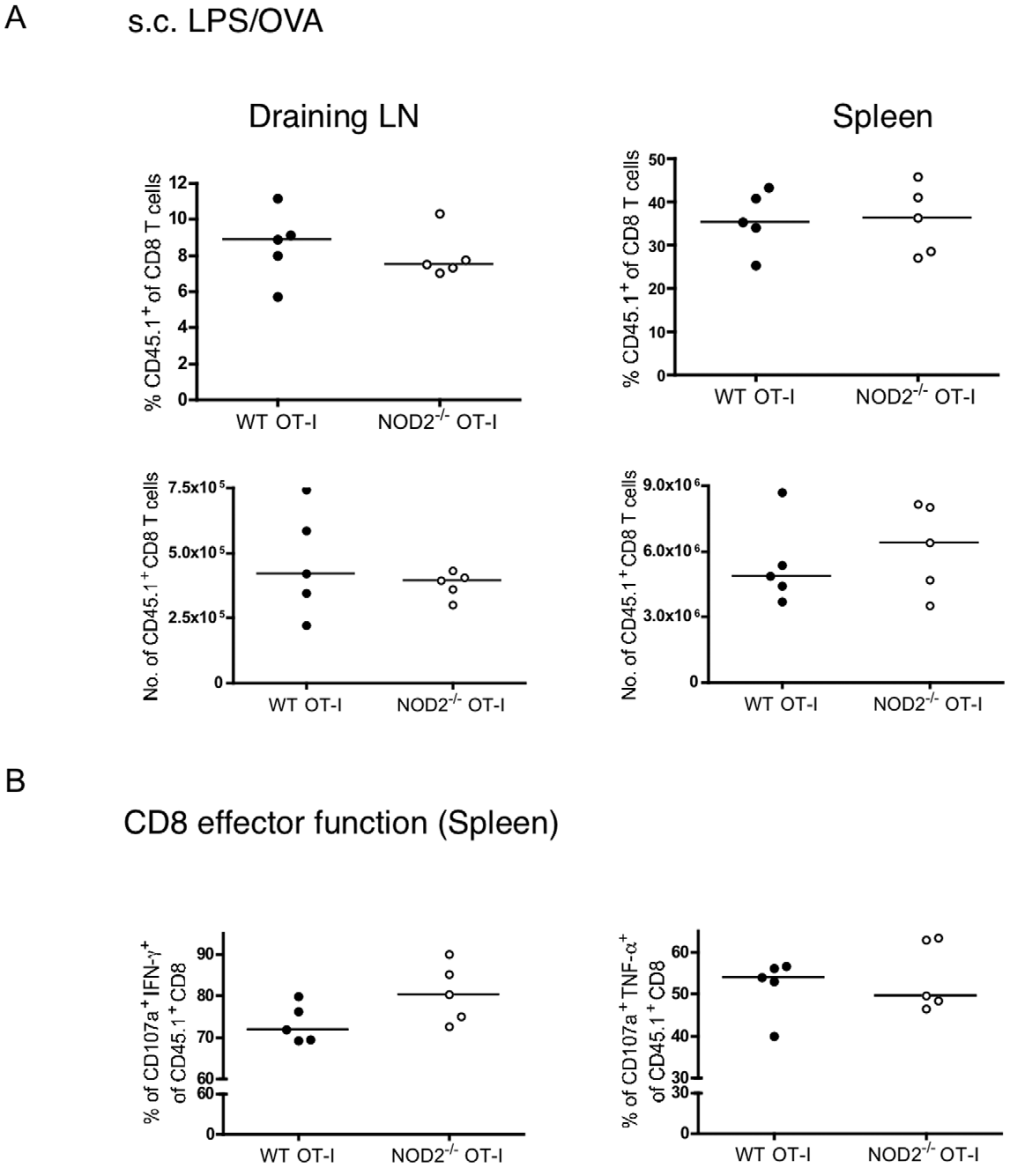
NOD2 is dispensable for TCR transgenic CD8 T cell expansion upon subcutaneous LPS/OVA immunization. 10^4^ purified CD8 T cells isolated from the spleens of CD45.1^+^ WT and CD45.1^+^ NOD2^−/−^ OT-I mice were injected i.v. to naïve WT mice, and one day later mice were injected with LPS and OVA protein s.c. (A) On day 6, mice were sacrificed and the percent of OT-I out of the CD8 T cell population as well as the total number of OT-I T cells recovered from the spleens and skin draining lymph nodes were analyzed. (B) The effector function of the transferred OT-I T cells was analyzed by CD107a, TNFα, and IFNγ staining following a 5-hour restimulation with 1 µM SIINFEKL peptide. The data in A and B are representative of 5 mice per group.

**Figure 6:**
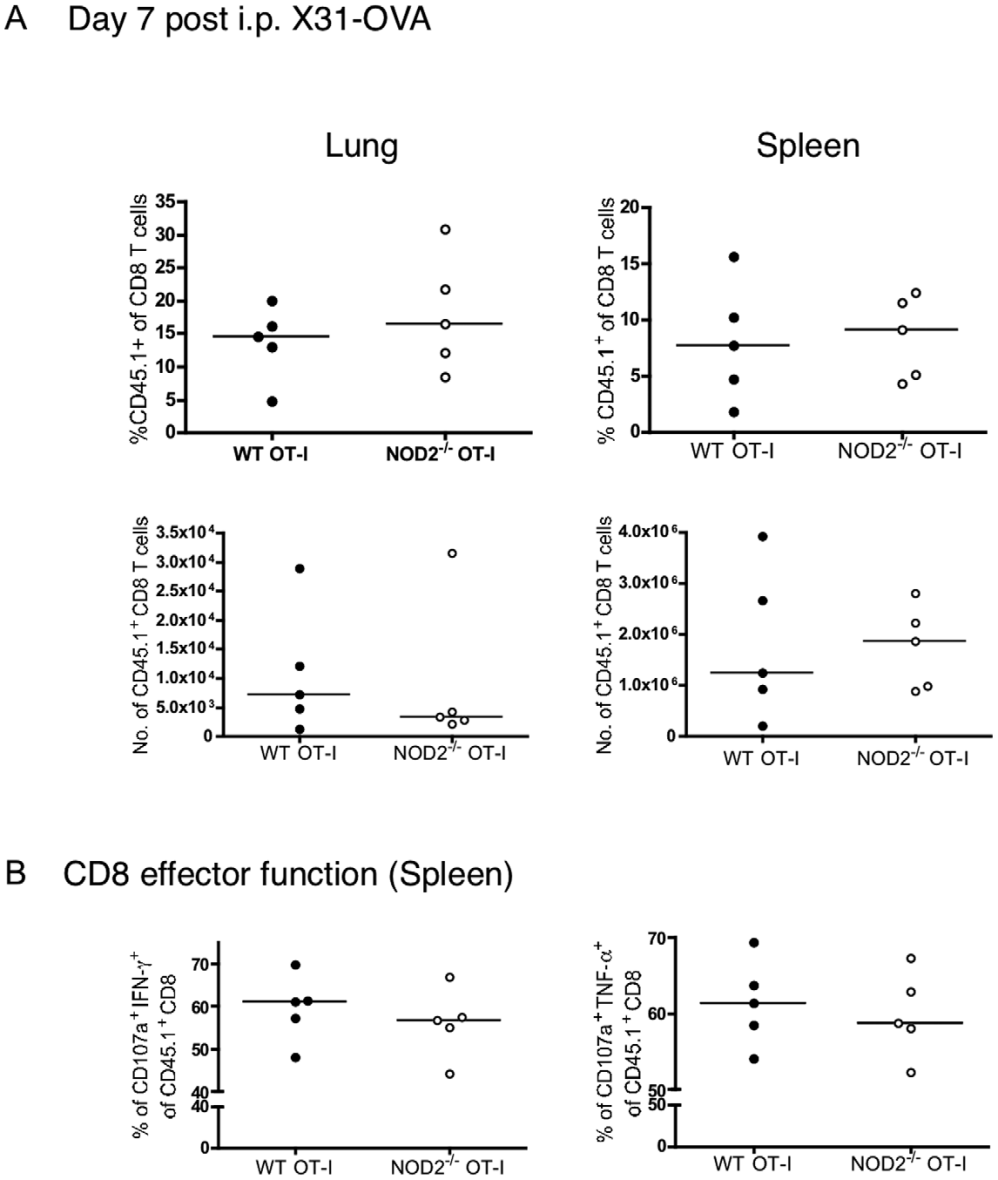
NOD2 is dispensable for TCR transgenic CD8 T cell expansion upon intraperitoneal influenza infection. 10^4^ purified CD8 T cells from CD45.1^+^ WT and CD45.1^+^ NOD2^−/−^ OT-I mice were injected i.v. to naïve WT mice, and one day later mice were infected intraperitoneally with influenza A/X31-OVA. (A) On day 7, mice were sacrificed and the percent of OT-I out of the CD8 T cell population as well as the total number of OT-I T cells in the lungs and spleens were analyzed. (B) The effector function of the transferred T cells was analyzed by CD107a, TNFα, and IFNγ staining following a 5-hour restimulation with 1 µM SIINFEKL peptide. The data in A and B are representative of 5 mice per group analyzed in a single experiment.

**Figure 7:**
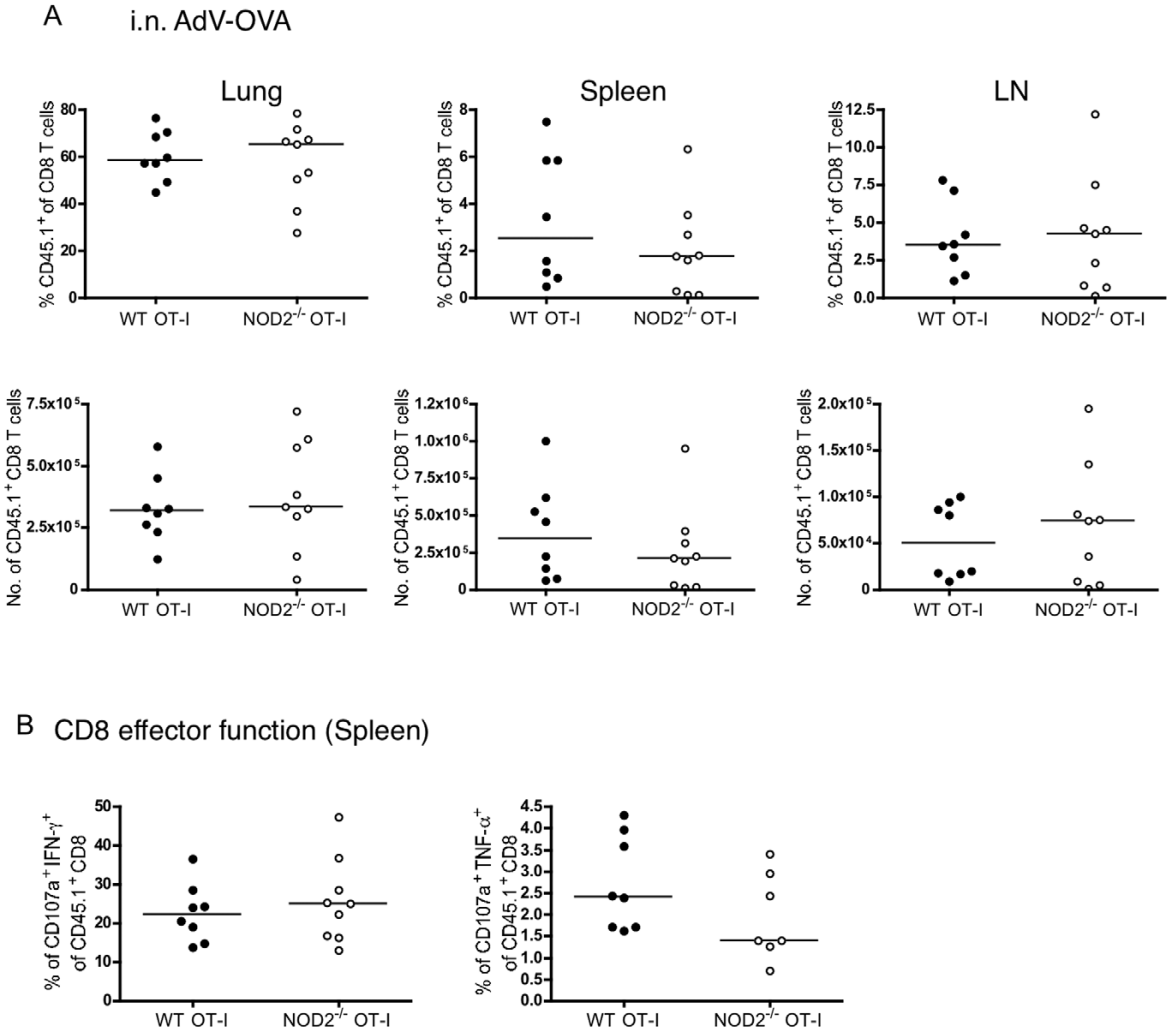
NOD2 is dispensable for TCR transgenic CD8 T cell expansion following intranasal delivery of adenoviral vectors. 10^4^ purified CD8 T cells from CD45.1^+^ WT and CD45.1^+^ NOD2^−/−^ OT-I T cells were injected i.v. into naïve WT mice, and one day later mice were treated with 10^9^ pfu Ad-OVA intranasally. (A) On day 10, mice were sacrificed and the percent of OT-I out of the CD8 T cell population as well as the total number of OT-I T cells in the lungs, spleens and mediastinal lymph nodes were analyzed. (B) The effector function of the transferred OT-I T cells was analyzed by CD107a, TNFα, and IFNγ staining following a 5-hour restimulation with 1 µM SIINFEKL peptide. The data in A and B are summary of two experiments, each with 4–5 mice per group.

We next investigated whether this 2-fold defect in the numbers of NOD2-deficient T cells following respiratory influenza infection was recapitulated upon stimulation in vitro or with other forms or routes of immunization. WT or NOD2^−/−^ OT-I T cells proliferated similarly to peptide stimulation, as measured by CFSE dilution, and similarly upregulated surface markers in response to anti-CD3 or anti-CD3/CD28 stimulation (Fig. 4). Thus in vitro, OT-I and NOD2^−/−^ OT-I T cells were indistinguishable in their response to antigen. Moreover, using the same adoptive transfer system, as shown in figure 2, we failed to observe any defect in CD8 T cell numbers or effector function of NOD2^−/−^ T cells after s.c. immunization with LPS/OVA, i.p. delivery of Influenza A X31, or i.n. delivery of a replication defective adenovirus carrying the SIINFEKL epitope (Fig. 5, 6, and 7).

### NOD2-deficient T cells that develop in the presence of extrinsic NOD2 show no defect in the immune response to respiratory influenza virus infection

**Figure 8:**
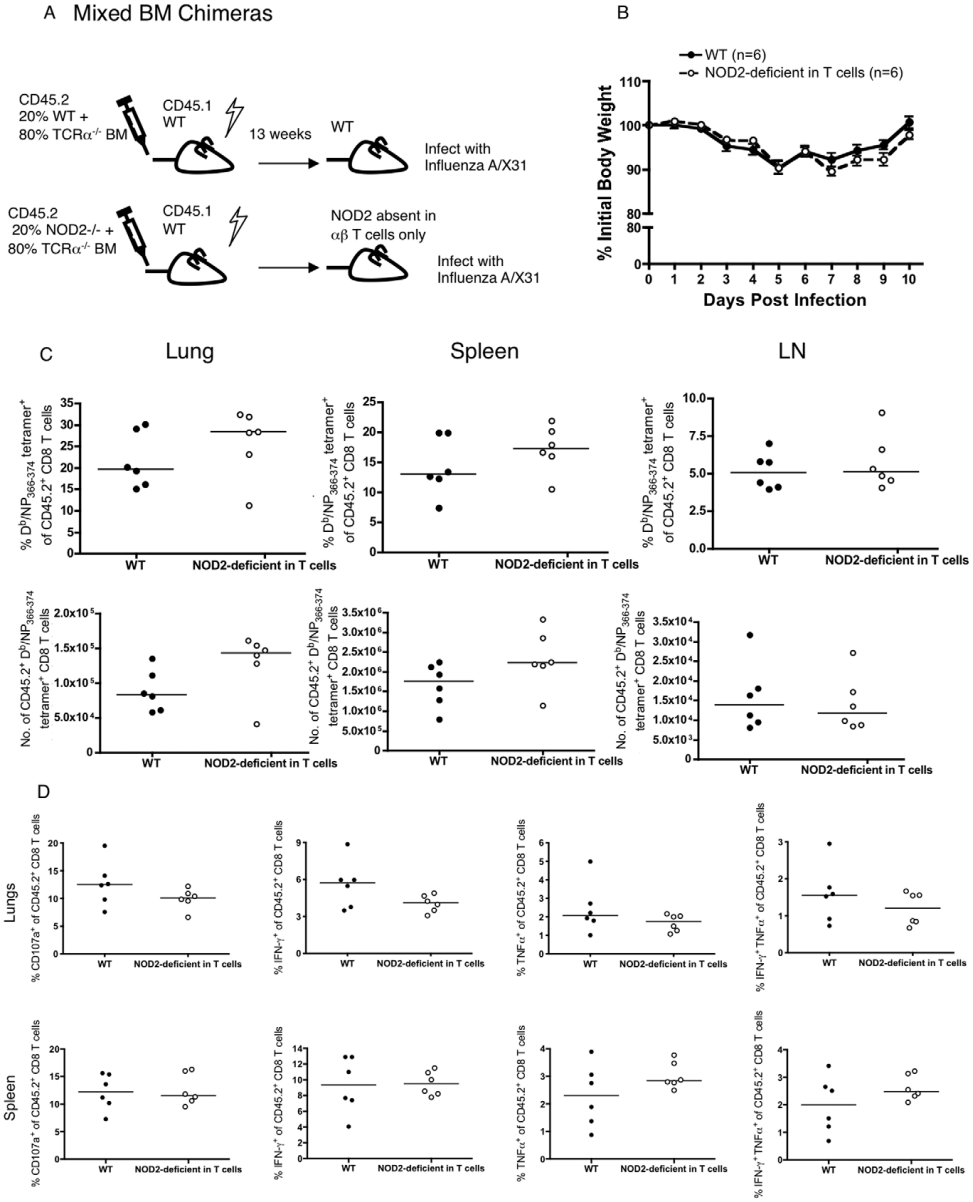
Mixed bone marrow chimeras fail to show a CD8 T cell intrinsic requirement for NOD2 in the mice. Mixed bone marrow chimeras were generated by reconstituting lethally irradiated CD45.1 C57BL/6 WT mice with a mixture of CD45.2 TCRα^−/−^ with WT or NOD2^−/−^ bone marrow cells as illustrated in panel (A). At 13 weeks post reconstitution, mice were infected intranasally with influenza A/X31. (B) Body weights of the mice following influenza infection are shown as the mean ± SEM for the 6 mice per group. (C) The percentage and the total number of endogenous CD45.1 NP_366–374_-specific CD8 T cells were measured using D^b^/NP_366–374_ tetramers on day 10 post-infection. (D) The effector function of influenza specific CD45.1 CD8 T cells in the lungs and spleens were analyzed by ex vivo restimulation with NP_366–374_ peptide at day 10 post infection. Results are shown for 6 individual mice per group, analyzed in a single experiment.

**Figure 9:**
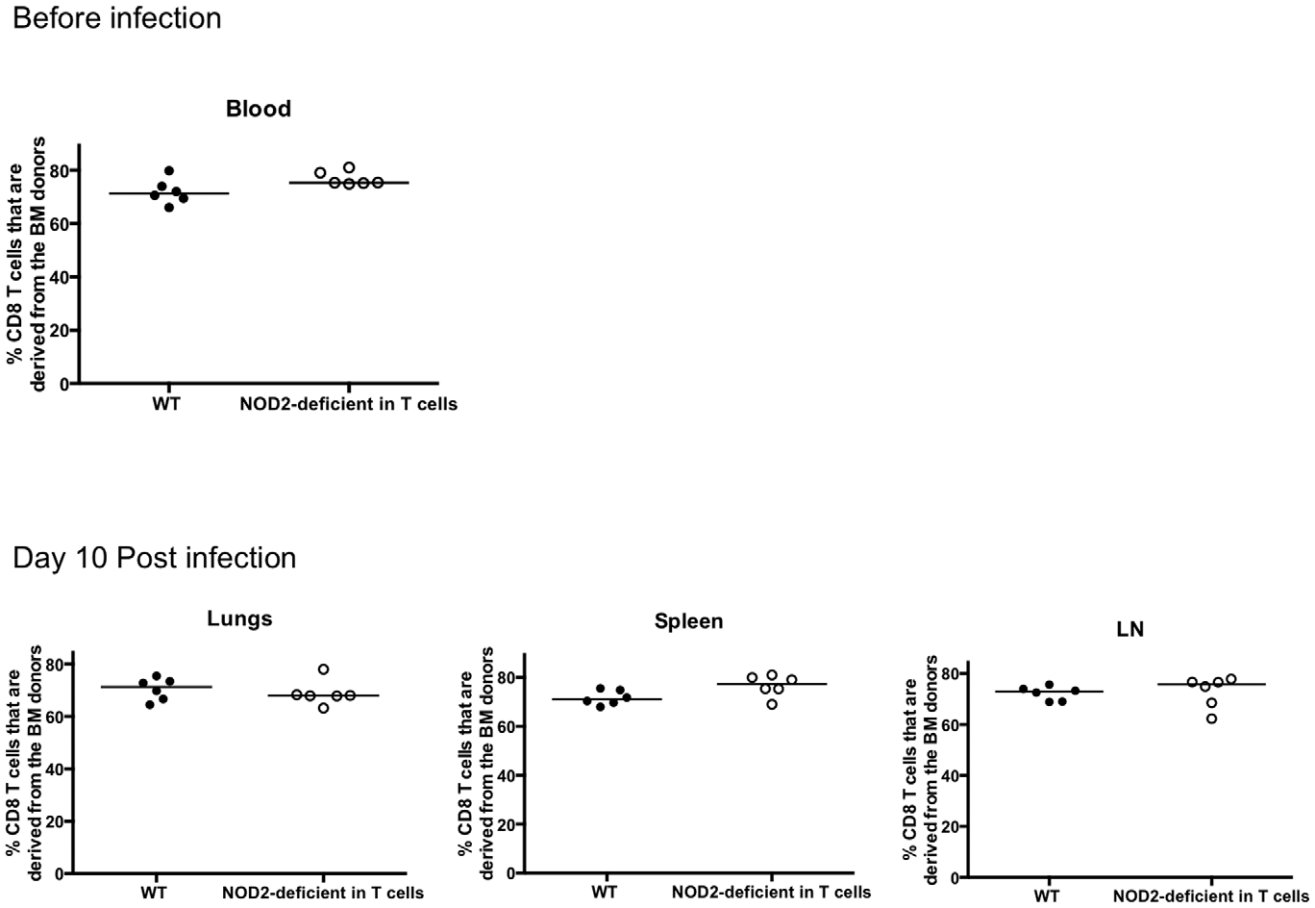
WT/TCRα^−/−^ or NOD2^−/−^/TCRα^−/−^ reconstituted mice show similar chimerism following reconstitution. The reconstitution efficiency of the CD8 T cells in the chimeras both before infection (blood) and at the time of harvest (day 10 post infection) was monitored by tracking the % of CD8 T cells expressing the congenic marker of the donor bone marrow (CD45.2).

As NOD2^−/−^ OT-I T cells were isolated from a mouse that lacks NOD2 in all cells, the experiments in Figure 2 do not differentiate between a requirement for NOD2 in the T cells during the immune response from a requirement for NOD2 expression in other cells during the development or maturation of the T cells prior to their initial antigen exposure. To address this issue, we generated mixed bone marrow chimeras in which only the bone marrow derived αβ T cells lack NOD2 and compared these to completely NOD2-sufficient mice (Fig. 8A), using a non-transgenic model. Following the generation of the mixed bone marrow chimeras, mice were rested for 3 months, before intranasal infection with influenza X31 (the parental virus from which the X31-OVA variant was derived [11]). The extent of chimerism was similar between mice before and after influenza infection (Figure 9). Consistent with the result obtained in Figure 2, NOD2 deficiency in the T cells did not impact on the overall weight loss of the mice (Fig. 8B). However, in contrast to the result obtained in Figure 2, which used adoptive transfer of WT or NOD2^−/−^ TCR transgenic T cells, in the non-transgenic bone marrow chimera experiments shown here, we found that the absence of NOD2 in the T cells alone had no impact on this endogenous CD8 T cell response (Fig. 8C and D). Thus, the T cells that have developed in the presence of NOD2 in other cells in the mouse are fully able to respond to a subsequent respiratory influenza virus infection.

## Discussion

These studies revealed a modest and selective defect in the numbers of expanded transgenic CD8 T cells recovered in the lung, spleen and LN following respiratory influenza infection when the T cells lack NOD2 during their development. However, no such defect was observed in polyclonal NOD2 deficient T cells that have developed in the presence of NOD2 in other cells. Moreover, this defect in the TCR transgenic model was not observed when the virus was delivered by the i.p. route of infection, in which influenza undergoes minimal replication. Neither was this defect observed when a non-replicating form of Ag (LPS/OVA or replication defective AdV-OVA) was used. Thus it appears that with the TCR transgenic model, some aspect of the response is sensitive to NOD2 when there is active viral replication in the lung, but this effect is lost with a non-replicating vector. The failure to recapitulate the CD8 T cell defect in the respiratory infection model in mixed bone marrow chimeras in which T cells lack NOD2, but other cells express NOD2, argues that T cell intrinsic NOD2 does not contribute to the CD8 T cell response even in the respiratory infection model. Thus we surmise that in the TCR transgenic model, the OT-I T cells that developed in the absence of NOD2, have a selective defect that leads to a 2-fold decrease in their accumulation during the primary immune response in vivo, a defect that is seen only under the specific conditions of a live respiratory viral infection, but is lost using non-replicating forms of Ag. The decreased numbers of NOD2^−/−^ OT-I T cells observed in the adoptive transfer model may therefore reflect a defect in the T cells due to their having developed or matured in the absence of extrinsic NOD2.

We tested several obvious candidates that might be important during viral infection, such as the type I IFN receptor, or the costimulatory receptors 4-1BB [12] and GITR [13] but did not find any defects in the CD8 T cells from uninfected NOD2^−/−^ as compared to WT mice before or after activation in vitro. Thus, the nature of this developmental or maturational defect in T cells that have developed in the absence of NOD2 in other cells, and that is only revealed under conditions of live respiratory viral infection remains to be determined. Based on our results, we speculate that the T cells that develop in the absence of NOD2 could have a selective defect in expression of a receptor or intracellular signaling molecule required for the response to a factor induced only with a replicating virus. As the defect observed is relatively modest (only a 2-fold deficit after 9 days of T cell expansion in the mice), the nature of this defect may be difficult to identify.

Regardless of the nature of this apparent developmental defect, the data presented here clearly show that NOD2 is not required intrinsically in the T cells for the CD8 T cell response to antigen under a variety of conditions in vitro and in vivo. These studies also illustrate a pitfall of using adoptive transfer models with gene-deficient T cells to explore T cell intrinsic defects in a pathway, as the T cells develop in the absence of that gene in all cells in the mouse. On the other hand, the mixed bone marrow chimeras allow the role of a particular gene to be assessed in the T cells in a context where they develop in the presence of that gene in all other cells and as such, in the absence of a cell type specific knockout, is the preferred method, as compared to adoptive transfer models, for evaluating cell specific gene deletion.
